# Quantitative evaluation of muscle synergy models: a single-trial task decoding approach

**DOI:** 10.3389/fncom.2013.00008

**Published:** 2013-02-26

**Authors:** Ioannis Delis, Bastien Berret, Thierry Pozzo, Stefano Panzeri

**Affiliations:** ^1^Robotics, Brain and Cognitive Sciences Department, Istituto Italiano di TecnologiaGenoa, Italy; ^2^Communication, Computer and System Sciences Department, Doctoral School on Life and Humanoid Technologies, University of GenoaGenoa, Italy; ^3^UR CIAMS, EA 4532 – Motor Control and Perception Team, Université Paris-Sud 11Orsay, France; ^4^Institut Universitaire de France, Université de Bourgogne, Campus UniversitaireUFR STAPS Dijon, France; ^5^INSERM, U1093, Action Cognition et Plasticité SensorimotriceDijon, France; ^6^Center for Neuroscience and Cognitive Systems @UniTn, Istituto Italiano di TecnologiaRovereto, Italy; ^7^Institute of Neuroscience and Psychology, University of GlasgowGlasgow, UK

**Keywords:** muscle synergies, reaching, arm movement, task decoding, single-trial analysis

## Abstract

Muscle synergies, i.e., invariant coordinated activations of groups of muscles, have been proposed as building blocks that the central nervous system (CNS) uses to construct the patterns of muscle activity utilized for executing movements. Several efficient dimensionality reduction algorithms that extract putative synergies from electromyographic (EMG) signals have been developed. Typically, the quality of synergy decompositions is assessed by computing the Variance Accounted For (VAF). Yet, little is known about the extent to which the combination of those synergies encodes task-discriminating variations of muscle activity in individual trials. To address this question, here we conceive and develop a novel computational framework to evaluate muscle synergy decompositions in task space. Unlike previous methods considering the total variance of muscle patterns (VAF based metrics), our approach focuses on variance discriminating execution of different tasks. The procedure is based on single-trial task decoding from muscle synergy activation features. The task decoding based metric evaluates quantitatively the mapping between synergy recruitment and task identification and automatically determines the minimal number of synergies that captures all the task-discriminating variability in the synergy activations. In this paper, we first validate the method on plausibly simulated EMG datasets. We then show that it can be applied to different types of muscle synergy decomposition and illustrate its applicability to real data by using it for the analysis of EMG recordings during an arm pointing task. We find that time-varying and synchronous synergies with similar number of parameters are equally efficient in task decoding, suggesting that in this experimental paradigm they are equally valid representations of muscle synergies. Overall, these findings stress the effectiveness of the decoding metric in systematically assessing muscle synergy decompositions in task space.

## Introduction

The question of how the central nervous system (CNS) coordinates muscle activity to produce movements is central to the understanding of motor control (Tresch et al., 1999). The human brain has to deal with a redundant musculoskeletal system comprising of approximately 600 muscles actuating approximately 200 joints. It has been suggested that the CNS reduces the complexity of this control problem by exploiting various types of modularity present in the motor system (Bizzi et al., 2002, 2008; Flash and Hochner, 2005; Berret et al., 2009). A prominent example of such modularity is given by muscle synergies (D'Avella et al., 2003; Ting and McKay, 2007), loosely defined as stereotyped patterns of coordinated activations of groups of muscles. According to this hypothesis, the muscle patterns driving movements originate from linear combinations of a small number of synergies presumably recruited by a premotor drive generated by some neuronal population (Delis et al., 2010; Hart and Giszter, 2010).

**Figure 1:**
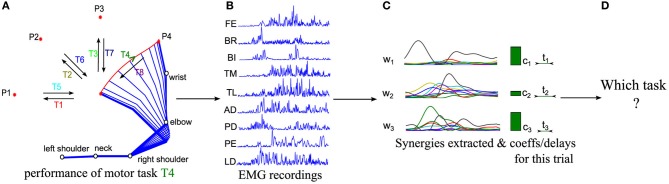
**Schematic representations of the logics of our procedure. (A)** Sketch of the kinematics of five joints during the execution of a center-out movement to target P4 (referred to as task T4 in the following). **(B)** EMG signals from 9 muscles are recorded in each trial with surface EMG devices during movement execution. **(C)** Muscle synergies are extracted from the whole dataset and the corresponding synergy parameters are computed for each trial. **(D)** We finally decode which motor task is performed in each trial from the extracted synergies and activation coefficients.

A relatively standard approach to individuate putative muscle synergies from EMG recordings of multiple muscles (Figure 1B) while subjects perform a variety of motor tasks (Figure 1A) is to first apply dimensionality reduction techniques to decompose the recorded EMGs into a set of synergies (Figure 1C), and then to assess validity of the decomposition using measures of goodness of approximation such as the Variance Accounted For (VAF) (D'Avella et al., 2006; Torres-Oviedo et al., 2006). This analytical approach has held valuable insights and hypotheses about the structure of modularity in muscle space.

Yet, actions are defined in task space and evaluation of the functional role of muscle synergies requires relating them explicitly to the execution of motor tasks (Todorov et al., 2005; Nazarpour et al., 2012; Ting et al., 2012). For this reason, the analytical framework needed to critically test synergy decompositions on empirical data should include additional elements that are currently not considered systematically. First, synergies must constitute not only a low dimensional but also a functional representation of a variety of motor tasks (Overduin et al., 2008). Thus, to evaluate how well muscle synergy recruitment relates to differences across motor tasks, we ought to quantify how well the executed motor tasks can be distinguished on the basis of the synergy activation coefficients (Brochier et al., 2004; Torres-Oviedo and Ting, 2010; Chvatal et al., 2011).

Second, the CNS generates appropriate motor behaviors on single trials, thus synergy recruitment must accurately describe single-trial muscle activations (Tresch et al., 1999; Torres-Oviedo and Ting, 2007; Roh et al., 2011; Ranganathan and Krishnan, 2012). Third, to evaluate the extent to which muscle synergies implement a dimensionality reduction, the number of synergies that discriminate among different task-related movements must be correctly identified and compared to both the degrees of freedom of the musculoskeletal system (e.g., number of muscles) and/or the number of tasks (Ting and McKay, 2007). The estimation of which synergies are needed to fully describe all the task-discriminating movement variations is therefore crucial. Moreover, muscle synergies can contribute to motor function even if their activations do not depend on the task at hand (e.g., if they relate to body posture or reflect biomechanical constraints). To understand the function of a putative set of synergies, it is important to be able to easily tease apart task-dependent and task-independent synergies.

In practice however, not only the identity of these synergies, but also their number and their contribution to task discriminating variations, is unknown a priori. To select the number of synergies, the most widely used criteria rely on the dependence of the amount of variance explained upon the number of synergies. However, criteria solely based on VAF depend substantially also on factors not related to task execution, such as noise (neural variability, measurement fluctuations etc.) and preprocessing (filtering, averaging of EMG signals etc.) and cannot distinguish synergy that describe task-to-task variations from synergies that do not. We argue that synergy model selection should reflect not only the reconstruction of the dataset but also include a way to assess the reliability of the associated mapping from synergy recruitment to motor task identification.

To address these needs, here we propose, implement and validate on EMG data a method (schematized in Figure 1) for predicting, on a single-trial basis, the motor task from the synergy activation parameters. The method is based on quantifying the task discriminability afforded by one or more synergies, and then using this for an automated objective selection of the minimal set of synergies containing all information about such salient task-related differences in synergy activation patterns. We validate the robustness and applicability of the method using simulated EMG datasets. We finally apply it to real EMG recordings during a reaching task to illustrate how this method may be used for individuating synergy sets relevant for the execution of a given set of tasks, and for evaluating in task space the effectiveness of different types of muscle synergy decompositions.

## Materials and methods

### Muscle synergy extraction

To identify muscle synergies, we used the time course of EMG activity of all recorded/simulated muscles in all individual trials for each task. We considered two well-established mathematical models for the representation of muscle patterns as synergy combinations: synchronous and time-varying.

#### Synchronous synergy model

We used the Non-negative Matrix Factorization (NMF) algorithm (Lee and Seung, 1999) to extract synchronous synergies. In this model, the EMGs are represented as a linear combination of a set of time-invariant activation balance profiles across all muscles activated by a time-dependent activation coefficient:

(1) $$m^{s}(t)=\sum_{i\;=\;1}^{N}c_{i}^{s}(t)w_{i}+\varepsilon^{s}(t)$$

where *m*^*s*^(*t*) is again the EMG data of all muscles at time *t*; *w*_*i*_ is the synergy vector for the *i*-th synergy; *c*^*s*^_*i*_(*t*) is the scalar coefficient for the *i*-th synergy at time *t*; *N* is the total number of synergies composing the dataset; and ε^*s*^(τ) is the residual (e.g., noise). *N* is an input to the NMF algorithm, so we varied the number of extracted synergies from 1 to 8. In this case, the sample-independent muscle synergies are time-invariant vectors and the parameters that have to be modified in each sample *s* are the time-varying waveforms *c*^*s*^_*i*_(*t*) (Cheung et al., 2005)-the superscript *s* is used to denote sample-dependent quantities.

#### Time-varying synergy model

We used the time-varying synergies model first introduced in (D'Avella and Tresch, 2002). According to it, a muscle pattern recorded during one sample *s* is decomposed into *N* time-varying muscle synergies combined as follows:

(2) $$m^{s}(t)=\sum_{i\;=\;1}^{N}c_{i}^{s}w_{i}(t-t_{i}^{s})+\varepsilon^{s}(t)$$

where *m*^*s*^(*t*) is a vector of real numbers, each component of which represents the activation of a specific muscle at time *t*; *w*(τ) is a vector representing the muscle activations for the *i*-th synergy at time τ after the synergy onset; *t*^*s*^_*i*_ is the time of synergy onset; *c*^*s*^_*i*_ is a non-negative scaling coefficient; and ε^*s*^(τ) is the residual (e.g., noise). This is a linear model providing a very compact representation of the muscle activity during one sample, since it has only two free parameters (one amplitude and one time coefficient) for each synergy (D'Avella et al., 2006). Note that the synergies *w*_*i*_ are sample-independent, whereas the parameters *t*^*s*^_*i*_ and *c*^*s*^_*i*_ must be adjusted for each sample (i.e., trial or task).

We fed the dataset to the time-varying synergies extraction algorithm to identify a set of muscle synergies and their activation coefficients that reconstructed the entire set of muscle patterns with minimum error. The number of extracted synergies *N* is a parameter of the model, and thus we repeated the extraction with a number of synergies ranging from 1 to 8. In order to minimize the probability of finding local minima, for each *N*, we ran the algorithm 10 times using different random initializations of the synergies and coefficients and selected the solution with the lowest reconstruction error. We used a convergence criterion of ten consecutive iterations for which the average error was decreased by less than 10^−6^. After extraction, the synergies (and the corresponding coefficients) were normalized to their maximum muscle activations.

#### Synergy similarity

We assessed the robustness of the synergy sets extracted from different experimental datasets as done in (D'Avella et al., 2003). In brief, we quantified the similarity between pairs of synergies as their correlation coefficient. For evaluating the similarity of the whole ensembles of synergies recorded in different datasets, we started by selecting the pair with the highest similarity, and then the synergies in that pair were removed from their sets. We then computed the similarities between the remaining synergies and repeated this procedure until all synergies in the smallest set had been matched. In the case of time-varying synergies, we computed the correlation coefficient of the two synergies over all possible delays and selected the delay that maximized similarity (see D'Avella et al., 2003 for details).

### Classical VAF-based criteria for assessing the validity of synergy decompositions and for selecting the smallest set of synergies

The purpose of this study is to develop a methodology to select, in a considered dataset, the smallest set of synergies that accounts for all task-discriminating variability in the recorded EMG dataset. Before we describe this new methodology, in this Subsection we briefly summarize the current methodology to choose the set of synergies used to decompose a dataset.

Synergy selection can be cast as a model selection problem, because different synergy decompositions are obtained when varying the number of extracted synergies. In the literature, there are typically *ad-hoc* criteria to assess the number of synergies, all based on the dependence of the amount of explained variance on the number of synergies extracted (*N*). This VAF is a measure of how well the actual muscle patterns can be fitted with a given set of synergies. The VAF of each synergy decomposition (D'Avella et al., 2006) is defined as follows:

(3) $$VAF=1-\frac{\sum_{s\;=\;1}^{S}\sum_{t\;=\;1}^{T}{\left\{m^{s}(t)-{\hat{m}}^{s}(t)\right\}}^{2}}{{\sum_{s\;=\;1}^{S}\sum_{t\;=\;1}^{T}\left\{m^{s}(t)-\bar{m}\right\}}^{2}}.$$

where *s* indexes samples and *t* indexes time steps; ${\hat{m}}^{s}(t)=\sum_{i=1}^{N}c_{i}^{s}w_{i}(t-t_{i}^{s})$ for the time-varying synergies and ${\hat{m}}^{s}(t)=\sum_{i=1}^{N}c_{i}^{s}(t){\text{w}}_{i}$ for the synchronous synergies model and $\bar{m}$ is the mean (over samples and time steps) activation vector.

To detect the correct number of synergies from the VAF, there are four main existing criteria all based on the “VAF curve,” i.e., the function quantifying the dependence of the VAF on the number of synergies extracted (*N*): (a) the point in the VAF curve at which a threshold (usually 0.9) is exceeded (“VAF-T” criterion) (Torres-Oviedo et al., 2006), (b) the point at which the highest change in slope (the “elbow”) is observed (“VAF-E”) (Tresch et al., 2006), (c) the point at which the curve “plateaus” to a straight line (“VAF-P”) (Cheung et al., 2005) and (d) the point at which any further increase in the number of extracted synergies yields a VAF increase smaller than 75% of that expected from chance (“VAF-S”) (Cheung et al., 2009b). VAF-S is implemented by first shuffling the EMG data across muscles and time steps and then extracting synergies from this “randomized” dataset. The VAF curve obtained from this decomposition exhibits an almost linear increase. Comparison of its slope with the slope of the real VAF curve at its point gives the selected number of synergies.

### New task-decoding based criterion for assessing the validity of the synergy decomposition and for selecting the smallest set of synergies

In this section, we present an additional method to quantitatively evaluate muscle synergies in task space and to automatically identify the smallest set of synergies that captures all the variance describing single-trial task-to-task variations in the dataset. To this aim, we first used a single-trial decoding analysis to quantify how well the single-trial coefficients of individual synergies or groups thereof discriminate between different tasks. A similar analysis has been implemented in the past in the context of muscle synergies (Brochier et al., 2004; Weiss and Flanders, 2004; Overduin, 2005) and in studies of modularity in the kinematics space (Santello and Soechting, 1998; Santello et al., 1998; Jerde et al., 2003). Intuitively, our reasoning is that VAF includes both the “interesting variance” (the one related to variations in synergy recruitment across tasks) and the “less interesting variance” (the one unrelated to variations in synergy recruitment across tasks and in some cases reflecting various sources of noise). In some condition, the presence of the latter variance may make difficult the selection of the correct number of synergies. Also, the removal of “noise” variance using VAF requires the experimenter's intuition and partly arbitrary or *ad-hoc* criteria (see section “Classical VAF-Based Criteria for Assessing the Validity of Synergy Decompositions and for Selecting the Smallest Set of Synergies” above). Our synergy evaluation method overcomes this problem by singling out (by single-trial task decoding) only the task-discriminating variance and then studying the dependence upon the number of synergies of this part of the variance.

#### Task decoding based metric

For a given set of synergies extracted via classical dimensionality reduction techniques (e.g., NMF), we defined the new metric as the decoding performance, i.e., the percentage of correct decoding of individual trials, based on the single-trial measure of their activation coefficients (or some parameters of them). To avoid inflating artificially the decoding performance because of data over-fitting, each trial was decoded based on the distribution of all other trials (decoding with leave-one-out cross-validation).

The task-decoding metric for determining the minimal set of synergies potentially depends on the choice of an algorithm used to decode the stimuli. Thus, to test the consistency of the results with respect to the details of the underlying calculations, we evaluated the performance of different decoding algorithms.

Here we used the following algorithms: (i) A linear discriminant algorithm (LDA), which worked as follows. For each pair of classes (i.e., motor tasks to be decoded), it first projects the *N*-dimensional values onto a hyperplane (i.e., a linear decision boundary) where the samples of each class are optimally separated. The direction of this line is the one that maximizes the ratio of the between-class over the within-class distances. Then, the trial to be predicted is assigned to one of the two classes by taking the one that has the minimum Euclidean distance in the direction of the decision boundary. An example of the decoding procedure using 2 pairs of synergies to decode the eight different motor tasks is given in Figure 6E. The LDA has determined the decision boundaries for classifying the trials to the motor task performed, and as a result, has separated the 2-dimensional space into eight regions, one for each task. Each point is assigned to the class represented by the colored region on which it lies. (ii) The quadratic discriminant (QDA), i.e., a discriminant algorithm that assumes unequal variances across classes leading to quadratic decision boundaries. (iii) The Naive Bayesian classifier (NB) which assumes that data points are independent with Gaussian in-class distributions and calculate the most likely class using Bayes theorem. (iv) The *k*-Nearest-Neighbors classifier with Euclidean distances (*k*-NN) (Duda et al., 2001). We set the number of neighbors *k* (a free parameter) to *k* = 10 because empirical investigations revealed that this selection maximized decoding performance for the dataset examined.

Unless otherwise stated, in this paper we relied on decoding using a LDA, because of its high computational speed and performance on the datasets considered here.

#### Automated procedure for selecting the minimal number of synergies based on task decoding

From the proposed decoding based metric, we developed an automated procedure to select the minimal number of synergies. This model selection technique is based upon the progressive evaluation of the statistical significance of the task-discriminating information added when progressively increasing the number of synergies in the decomposition model. After evaluating decoding performance with *N* = 1 synergy, the number of synergies in the decomposition model increases step by step, until the increase of synergies does not gain any further statistically significant increase of decoding performance. The procedure is automatic because of this statistical test of significance. In this way, the chosen set of *N* synergies is the smallest decomposition that captures all available task-discriminating variance within the synergy space.

**Figure 2:**
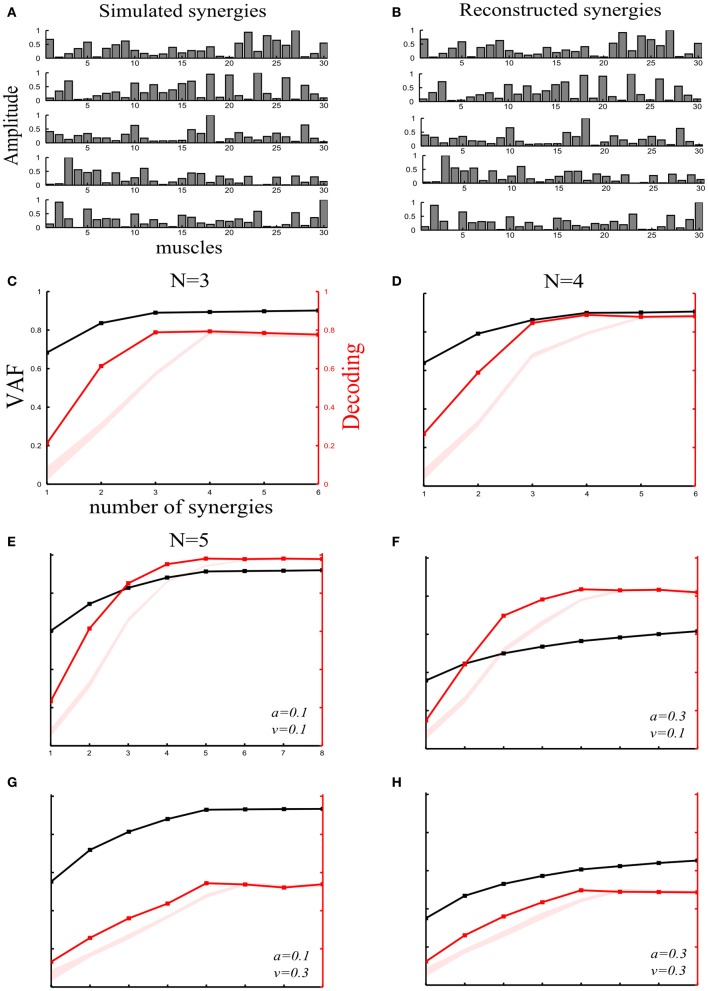
**Robustness of our method applied to synchronous synergies when varying the number of “ground-truth” synergies and the sources and levels of noise. (A)** Set of five simulated synchronous synergies used as the “ground-truth” for testing our method. **(B)** The five synergies recovered when the NMF algorithm was applied to the simulated EMG data. In all cases, the original synergies were accurately reconstructed. **(C–E)** VAF (black curves whose scale is indicated in left *y*-axes) and decoding performance curves (red curves whose scale is indicated in right *y*-axes) for datasets generated from 3**(C)**, 4**(D)**, and 5**(E)** “ground-truth” synergies. For noise levels, we used the “reference” values (*a* = 0.1 and *v* = 0.1). The red shaded areas represent the 5–95% confidence intervals of the bootstrap test for decoding. **(F–H)** VAF and decoding performance curves [plotted with the same conventions as in panels **(C–E)**] when generating data from five synergies and varying the simulated noise parameters as follows: increased signal-dependent noise (*a* = 0.3, *v* = 0.1) **(F)**; increased trial-by-trial variability of the activation coefficients (*a* = 0.1, *v* = 0.3) **(G)**; increased signal-dependent noise and trial-by-trial variability of the activation coefficients (*a* = 0.3, *v* = 0.3) **(H)**.

Crucial to this selection procedure is the test to determine if adding one more synergy significantly increases decoding performance. We needed to ensure that the different dimensionality of two models with *N* and *N* + 1 synergies does not lead to any artifactual difference in the computed percent correct values. Thus, we designed the statistical test as follows. For a given value *N*, we compare the decoding performance of the synergy parameters when using the *N* synergies with the decoding performance of the parameters of all subsets consisting of *N* − 1 synergies plus the parameters of the *N*-th synergy pseudo-randomly permuted (“shuffled”) across conditions. We repeat this shuffling procedure a number of times (100 in our implementation) to obtain a non-parametric distribution of decoding performance values in the null hypothesis that the additional synergy does not add to the decoding power of the synergy decomposition. In the following we evaluated this significance at the *p* < 0.05 threshold. The statistical threshold for significant increase of decoding performance was graphically highlighted in the plots of the decoding performance (% correct) as a function of the number of synergies as a shaded area indicating the 95% confidence intervals constructed using this bootstrap procedure (Figure 2C). In this way, if the original decoding performance curve enters in the shaded area at the *N*-th synergy, the *N*-th dimension does not increase significantly the decoding performance and therefore this suggests that *N* − 1 synergies should be selected. The selected number of synergies can be simply visualized as the smallest value of *N* for which decoding performance lies above the no-significance (shaded) area.

#### Choice of the synergy coefficients' parameters

In practice, task decoding is applied in synergy space, thus it requires working with synergy activation variables. The nature of such activation coefficients depends on the synergy model considered. After the decomposition into *N* synergies, the pattern of synergy activation in each trial can be described by a set of scalar parameters, vectors and temporal waveforms. To illustrate the method clearly, we first restricted our analysis to only one single-trial parameter per synergy for both models (time-varying and synchronous synergies), so each trial was represented by *N* values. The time-varying synergy model has two single-trial parameters (one scaling coefficient and one time delay) per synergy. We used the scaling coefficients which were shown to be more task-informative than the time-delays (see section “Results”). In the case of the synchronous model, we had to extract the single-trial parameters from the time-varying activation coefficient. We decided to use the time integral of the activation coefficient over the entire task period because preliminary investigations (not shown) revealed that integral measures largely outperformed the decoding performance obtained with other measures based on single points such as timing and amplitude of first or second activation peak (Karst and Hasan, 1991; Flanders et al., 1996) and measures based on Principal Component Analysis of the activation time course (Optican and Richmond, 1987). The use of the integrated EMG activity is standard and has been extensively used in biomechanical studies (e.g., Winter, 2009).

Then, we went on to investigate whether taking more single-trial parameters into account would increase the task identification power of each type of synergy decomposition. For the time-varying model, we evaluated the decoding performance using both single-trial parameters (scaling coefficients and time delays). For the synchronous model, the time-varying activation coefficients of the synergies add a large number of extra parameters (up to an independent parameter per time point if activation varied fast enough) that could potentially carry more task-discriminating information. To test for the possibility that complex multi-parameter single descriptions of the time course of activation of synchronous synergies carry more information, we progressively refined the parameterization of the single-trial activation coefficients by binning them in smaller bins and we computed the decoding performance as a function of the number of bins.

### EMG recording procedures

To validate the analytical methods developed here, we applied them to a set of EMGs recorded during the execution of a reaching task, as described in the following.

Four healthy right-handed subjects (AM, AB, AK, and ES) participated voluntarily in the experiment. The experiment conformed to the declaration of Helsinki and informed consent was obtained from all the participants, which was approved by the local ethical committee ASL-3 (“Azienda Sanitaria Locale,” local health unit), Genoa. The protocol consisted in executing reaching multijoint movements (flexions and extensions of the shoulder and elbow joints) in the horizontal plane (Figure 1A). The subjects sat in front of a table and were instructed to perform fast one-shot point-to-point movements between a central location (P0) and 4 peripheral locations (P1-P2-P3-P4) evenly spaced along a circumference of radius 40 cm. Subjects were supporting the weight of their arm by themselves; no device was used to remove static gravitational effects. The upper trunk was not restrained, but analysis of the kinematics data showed that its movement during the investigated tasks was negligible. The subjects made center-out (fwd) and out-center (bwd) movements (Figure 1A) to each one of the 4 targets. In total, the experimental protocol specified 8 distinct motor tasks denoted by T1,…,T8, each one of which was performed 40 times, thus the entire experiment consisted of 8 tasks × 40 trials = 320 samples. The subjects were asked to perform such a relatively high number of repetitions of each task because this was useful for the validation of the single-trial algorithms and of the impact of trial-to-trial variability on the generation of muscle activation patterns. This experimental design can be viewed as a variant of the one proposed in (D'Avella et al., 2006, 2008) since performance of each one of the 8 motor tasks requires executing movements to 8 different directions. The main difference with respect to this previous work is that instead of considering movements from the center of a circle to 8 points on its circumference, we analyze forward and backward movements between the center and 4 of these points. The order with which the movements were performed was randomized.

Electromyographic (EMG) activity was recorded by means of an Aurion (Milano, Italy) wireless surface EMG system. The EMG signals were recorded with a sampling rate of 1 kHz from the following muscles: (1) finger extensors (FE), (2) brachioradialis (BR), (3) biceps brachii (BI), (4) triceps medial (TM), (5) triceps lateral (TL), (6) anterior deltoid (AD), (7) posterior deltoid (PD), (8) pectoralis (PE), (9) latissimus dorsi (LD) (Figure 1B). The EMGs for each trial were digitally full-wave rectified, low-pass filtered (Butterworth filter; 20 Hz cutoff; zero-phase distortion), their duration was normalized to 1000 time steps and then the signals were integrated over 20 time-step intervals yielding a final waveform of 50 time steps. This is a standard EMG treatment in muscle synergy studies (see D'Avella et al., 2006). Body kinematics was recorded by means of a Vicon (Oxford, UK) motion capture system with a sampling rate of 100 Hz. Six passive markers were placed on the fingertip, wrist (over the styloid process of the ulna), elbow (over the lateral epicondyle), right shoulder (on the lateral epicondyle of the humerus), back of the neck and left shoulder. The kinematics data were low-pass filtered (Butterworth filter, cut-off frequency of 20 Hz) and numerically differentiated to compute tangential velocity and acceleration. Movement onset and movement end were identified as the times in which the velocity profile of the fingertip superseded 5% of its maximum. The mean movement duration varied across subjects from 370 to 560 ms. We verified that for all the subjects included in this analysis none of the muscles showed a systematic change in signal amplitude across the recordings sessions, which would be an indication that the EMG sensors were partially detached from the skin.

### Generation of simulated EMG datasets

To demonstrate the validity of our method and investigate its robustness, we tested it on simulated single-trial EMG responses in which the “ground truth” about which synergy set actually generated the data was known by construction, and that contained physiologically plausible sources of single-trial variance. We constructed two synthetic datasets by linear summation of a small number (from 2 to 5, depending on the simulation) of either synchronous or time-varying synergies and corrupted them with different physiologically plausible sources of single-trial variance: motor noise on the synergy activations and additive signal-dependent noise. The amount of each type of noise was parameterized by two separate free parameters: *v* and *a* respectively.

The first EMG dataset (simulation of synchronous synergies) was generated as a weighted combination of set of synchronous synergies according to Equation 2. The data simulated the activation of *M* = 30 muscles used for executing *R* = 40 repetitions of each one of *T* = 15 motor tasks, i.e., 40 × 15 = 600 simulated samples in total (Figure 2A). The *M*-dimensional synergies were drawn from exponential distributions with mean 10 were then normalized to each synergy's maximum muscle activation (Tresch et al., 2006). Their number was varied from *N* = 3 to 5 and their corresponding activation coefficients were assumed time-invariant for simplicity (Tresch et al., 2006). The use of time-invariant activation coefficients is common in the synchronous synergy framework (Ting and Macpherson, 2005; Torres-Oviedo et al., 2006). Typically, such time-invariant activations result from the preprocessing of the EMG signals to reduce dimensionality in the time domain (e.g., time-averaging of the muscle activities). However, the model can readily incorporate temporal variability too. In our simulations, we assumed that, to execute a motor task, each one of the *N* synergies was activated by a scalar coefficient drawn from a uniform distribution in the [0,1] interval. So, each task was represented as an *N*-dimensional vector of activation coefficients. The variability in the neural motor command, which in turn leads to trial-to-trial variations of the synergy coefficients in each task, was modeled as additive white Gaussian noise with covariance matrix *v*^2^**I**. Hence, we varied the parameter *v* to modulate the trial-to-trial variability of the synergy recruitment for each task.

**Figure 3:**
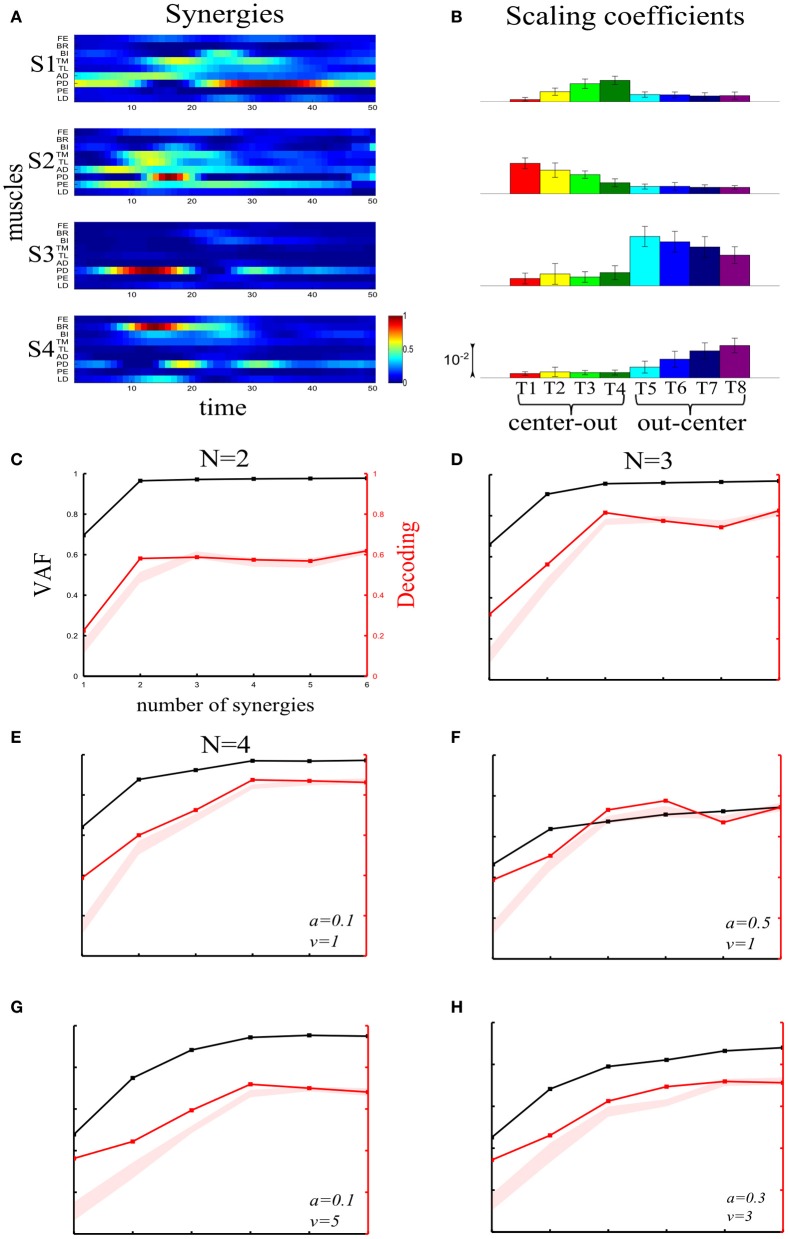
**Robustness of our method applied to time-varying synergies when varying the number of “ground-truth” synergies and the sources and levels of noise. (A)** The four time-varying synergies obtained from experimental recording during arm reaching as four matrices each representing the activity of 9 shoulder and elbow muscles over 50 time steps. This set of synergies was used to generate the simulated data used to test the method on time-varying synergies. **(B)** Histograms of the coefficients activating the four synergies across the 8 motor tasks performed. Histograms are plotted as means ± SDs across all trials to the given task. **(C–E)** VAF and decoding performance curves for datasets generated from 2**(C)**, 3**(D)** and 4**(E)** “ground-truth” synergies. For noise levels, we used the “reference” values (*a* = 0.1, *v* = 1). **(F–H)** VAF and decoding performance curves when generating data from four synergies and varying the simulated noise parameters as follows: increased signal-dependent noise (*a* = 0.5, *v* = 1) **(F)**; increased trial-by-trial variability of the activation coefficients (*a* = 0.1, *v* = 5) **(G)**; increased signal-dependent noise and trial-by-trial variability of the activation coefficients (*a* = 0.3, *v* = 3) **(H)**. Conventions as in Figure 2.

To construct the second simulated dataset (simulation of time-varying synergies), we used the time-varying synergies (from *N* = 2 to 4) and the corresponding scaling coefficients extracted from the single-trial experimental data of the typical subject (Figures 3A,B). Importantly, this simulated dataset was built such that, without any source of task-unrelated variability, the underlying set of synergies is theoretically sufficient to produce the EMG patterns of 9 muscles for the execution of 8 motor tasks and such that, even with other sources of noise added, all task-discriminating variations are described by the scaling coefficients of the “ground-truth” synergies. We computed the means of the extracted coefficients for each motor task and fitted their distributions with *N*-dimensional Gaussians. Based on these data, we modeled the motor noise that corrupts the coefficients and delays as a Gaussian whose standard deviation (SD) is a fraction *v* of the SDs measured on real EMGs. A value *v* = 1 corresponds to the amount of variability empirically found on real data, whereas *v* > 1 corresponds to trial-to-trial synergy recruitment variability larger than that of the experimental data. From the resulting Gaussian distributions, we generated 40 sets of activation coefficients *c*^*s*^_*i*_ per task which scaled the extracted synergies **w**_**i**_(t) according to Equation 1 to generate the simulated EMG dataset consisting of 9 muscles and 40 trials × 8 tasks = 320 samples. The duration of each sample was 50 time steps.

**Figure 4:**
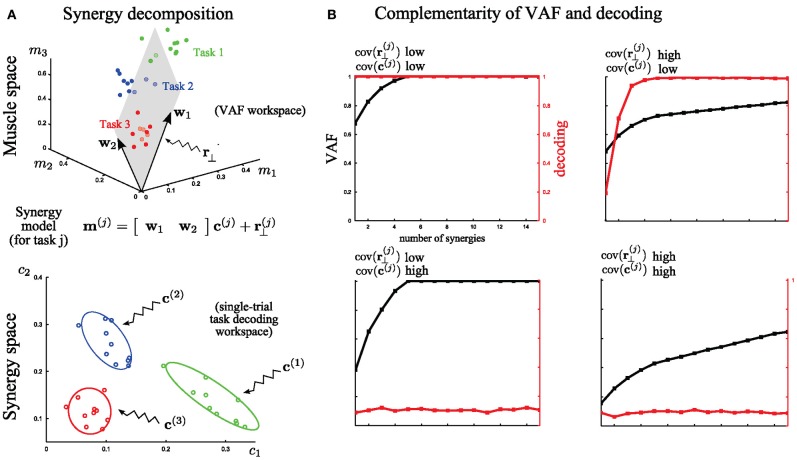
**A simple illustration of problems faced in assessing the quality of muscle synergy models. (A)** Top: Identification of 2 muscle synergies (***w***_***1***_ and ***w***_***2***_) from the activities of 3 muscles executing 3 motor tasks. The 3-dimensional muscle space is approximated by a linear 2-dimensional synergy space. Bottom: Distributions of the synergy activation coefficients across trials for the three tasks. The variability of these distributions determines how reliably synergy recruitment maps onto task accomplishment. **(B)** Illustration of the behavior of VAF (black curves and left *y*-axes) and decoding performance (red curves and right *y*-axes) as a function of the number of extracted synergies under four cases of extreme (either high or low) levels of variability. The dataset was generated by combining five synchronous synergies. These examples indicate that the two metrics assess the role of two different types of variance in the dataset.

For both simulations, the resultant data were then corrupted by task-unrelated variability during movement execution. This was done by adding the noise term ε^*s*^(*t*) in Equations 1, 2. Muscle activation patterns have been reported to be corrupted by noise that scales with the amplitude of the motor signal, termed as signal-dependent noise (Tresch et al., 2006). Such noise whose SD is proportional to the amplitude of the noiseless EMG was added on each muscle's simulated EMG activity. In summary, the two types of variability included in the generation of the simulated data set are:

- Additive noise (i.e., trial-to-trial variability) on the activation coefficients and delays (free parameter *v*). This is a motor noise that affects the synergy recruitment itself. This variability corresponds to the one depicted thereafter in Figure 4A bottom.
- Signal-dependent noise, i.e., additive noise whose SD is proportional to the magnitude of the noiseless activation pattern of an individual muscle *m* (σ^(*m*)^ = *a*^(*m*)^ ∑^*N*^_*i* = 1_ *c*_*i*_**w**^(*m*)^_*i*_(*t*), free parameter *a*). This noise affects all the muscles independently and could typically represent variability arising from motor neurons activity, but also noise related to signal pre-processing and measurement. This type of variability will generally affect dimensionality of the data set and corresponds to the one depicted thereafter in Figure 4A top (actually, it is even more general because it is not necessarily orthogonal to the synergy space).

The above simulation parameters were varied to test the robustness of the proposed method to all sources and different levels of noise. In all cases, the maximal values of these parameters were chosen as the largest values that allowed accurate reconstruction of the original synergies by the synergy extraction algorithms.

## Results

### Complementarity of VAF and decoding metrics for assessing the validity of synergy decompositions

Before we proceed to a detailed validation of the newly proposed decoding metric, we begin discussing and exemplifying the complementarity between our decoding metric and the traditionally used VAF metric (Ting and Chvatal, 2011). This is useful to both illustrate our methodology and justify the need for complementing the current evaluation of muscle synergy models with a task-space metric.

For this illustration, we simulated the activity of three muscles executing three different tasks (Figure 4; red, blue, green). For simplicity, we assume that muscle activation is described by a time-invariant scalar in this preliminary example. In each trial, the activation of these three muscles can be represented as a point in the 3-dimensional muscle space [as done in (Tresch et al., 2006) in simulations and in (Torres-Oviedo et al., 2006; Torres-Oviedo and Ting, 2007) with real data]. In Figure 4A (top), we show a total of 30 sample points (10 for each task). Muscle synergy identification consists in finding basis vectors spanning a lower-dimensional linear space in which the original data can be accurately described: in this case we extracted two synergies with non-negativity constraints (the arrows ***w***_1_ and ***w***_2_) that approximate at best the data by a 2-dimensional hyperplane defined by the 3-dimensional synergy vectors (Figure 4A top). This representation is considered good if the approximation error (i.e., the residual error *r*^(*j*)^_⊥_), defined as the distance of the data points from the hyperplane, is low. The currently most exploited metric to determine the set of synergies best describing a dataset is the VAF measure, which quantifies the proportion of variability in the EMG data set that is accounted for by the muscle synergy decomposition (see Equation 3 in section “Materials and Methods” for the exact definition). This metric is defined in muscle space. Thus, a high value of VAF means a high quality of reconstruction of the original muscle pattern (i.e., a relatively low *r*^(*j*)^_⊥_). In this part of the analysis, unsupervised learning only is performed and the available knowledge about the task is ignored.

However, as outlined in section “Introduction,” VAF alone cannot tell if, or how well, activations of these synergies describe variations of movement across different tasks in individual trials, and if two different synergies describe a different or the same type of task-discriminating variations. In other words, there is a second and equally important type of variability in the synergy decomposition that cannot be addressed by the VAF, which concerns trial-by-trial differences in the recruitment of muscle synergies. To analyze this, we move to coordinates in the synergy space (Figure 4A bottom). This variance reflects the spread of the synergy activation coefficients *c*^(*j*)^ for the execution of the same task. The extent to which this latter variance affects task discrimination (and thus likely also task execution) can be assessed by quantifying the identifiability of the task performed on every trial in the synergy space. This is because executing a motor task using muscle synergies requires a well-defined mapping from synergy recruitment to task identification (i.e., one possible outcome associated with a given synergy activation). To quantify the reliability of this mapping on a single-trial basis we introduced (see section “Task Decoding Based Metric”) the decoding metric which measures the percentage of times the task *j* is correctly predicted by the single-trial activation coefficients *c*^(*j*)^. This additional metric is necessary for the evaluation of muscle synergy representations with respect to task identification.

We further illustrate in Figure 4B the *complementarity* of the two metrics (VAF and decoding based) by considering EMG datasets corrupted with extreme levels (either high or low) of both types of variability described above. These datasets were generated from the five synchronous synergies shown in Figure 2A (see section “Materials and Methods” for details) and in all cases the noise levels were restricted so that the original synergies could be recovered from the synergy extraction algorithm (Figure 2B). Note that with very large noise, the original synergistic structure can be hidden or lost and therefore the extraction algorithm may fail to extract the correct synergies. Therefore, our analysis relies on the assumption that NMF's algorithms were able to recover the correct synergies (at least for the correct *N*, which was checked a posteriori).

First, we considered low levels of both types of variance for each task (the variance in muscle space, assumed to lie on a plane that is orthogonal to the synergy space, which characterizes how the actual muscle pattern varies across trials and the variance in synergy space, which characterizes trial-to-trial variations in the synergy recruitment). Note that the distinction between these two sources of variability is not unrealistic as it may reflect actual neural noise acting at different levels of the CNS. With low variance, the VAF metric reveals that five synergies reconstruct accurately all the muscle patterns and the decoding metric validates that all the tasks are perfectly discriminable from the activation coefficients of these synergies (Figure 4B, top left). Then, we tested the case of very high variance only in *r*^(*j*)^_⊥_. Because such variability cannot be written as synergy decomposition (by hypothesis), the VAF by the extracted synergy decomposition is never sufficiently high: the high levels of unstructured noise do not allow a good approximation of the data in a low-dimensional space. Nevertheless, decoding performance proves that 5 synergies describe all task-to-task differences; therefore these synergies constitute the minimal set of synergies that guarantees a reliable mapping between their activation and task identification (Figure 4B, top right). On the other hand, when there is a very high variability in the synergy activations *c*^(*j*)^ then the synergy decomposition cannot distinguish between different tasks even though the dimensionality reduction is not affected. This is shown by the decoding performance curve that never exceeds significantly chance level. Hence, although all the data points still lie on a 5-dimensional space (*VAF = 1* exactly for *N* = 5), there is no way to guarantee what task will be executed at fixed activation of the synergies (in this extreme case, the mapping is even a uniform random variable –chance level) (Figure 4B, bottom left). Finally, we consider high variance for both *r*^(*j*)^_⊥_ and *c*^(*j*)^ simultaneously. As expected, VAF exhibits no saturation point and decoding performance is at chance level. So, in this case, the identified synergy decomposition neither reconstructs well the original (i.e., recorded) muscle activities nor discriminates between tasks (Figure 4B, bottom right). This “worst case” scenario would typically invalidate the synergy decomposition because both metrics yield low scores.

These extreme but theoretically plausible cases exemplify well the usefulness of a systematic methodology that evaluates quantitatively not only the approximation of the muscle space by the synergy space (addressed by the VAF) but also the mapping between synergy activation and task identification (addressed by single-trial task decoding). Such an approach will allow an objective and automatic assessment of muscle synergy models/decompositions and thus, it can serve as a model selection criterion.

It is useful to consider other possible conceptual cases in which concurrent evaluation of VAF and task decoding may provide valuable insights. First, consider a case when VAF individuates a larger set of synergies than that individuated by decoding. The extra synergies identified by VAF explain a useful amount EMG variance but do not add any task-discriminating information. This may happen either because such extra synergies are activated always in the same way in all tasks (something that can be tested by verifying that when considered individually they lead only to random task decoding) or that they do not add any task discrimination to that already carried by the minimal set of synergies determined by the decoding metric. Careful decoding analysis of the individual extra synergies contributing to VAF but out of the minimal task-decoding set may be useful to individuate synergies (e.g., postural synergies) that may be important for generating the appropriate movements even if their activation is constant across tasks. A second possibility is that decoding selects a larger set of synergies than VAF. This case indicates that some synergies (because e.g., they have little signal amplitude) do not explain a large part of the variance of the dataset, yet they provide unique information about task-to-task differences not carried even by other synergies in the set with larger amplitude. These considerations suggest the potential value and complementarity of the insights provided by the joint use of VAF and decoding metrics.

In the following, we aim to show how our new method is crucial in this respect. We will illustrate how the VAF and decoding curves behave when muscle synergies are extracted from real data and verify that the method is reliably applicable to a variety of realistic EMG datasets with different properties giving robustly correct results.

### Simulation studies of the robustness of the decoding-based method

To demonstrate and test our novel method for selecting the set of synergies that describe all task-related differences at best, we used two types of simulated datasets (one for each type of muscle synergy model, see section “Materials and Methods” for details). Importantly, in both simulated datasets, which contain both task-discriminating and non-discriminating (“noise”) variance, we know by construction the number of synergies sufficient to describe the entire task-discriminating part of the EMG variance. This allows a direct evaluation of the performance of the method against a ground truth. Moreover, by manipulating the parameters (such as the level of noise, or the number of true synergies, or the number of samples recorded), we can investigate how these variables affect the quality of synergy detection. In the following, we first validate the methodology by implementing various tests on the dataset generated by synchronous synergies and then show that its application extends naturally to the time-varying synergy model.

#### Assessment of the method's performance when applied to synchronous synergies

***Varying the number of “ground truth” synergies.*** First, we checked the extent to which our method could identify the correct set of synergies that generated the data, by varying the number of ground-truth synergies from which we generated the simulated EMG data. More precisely, we simulated three, four or five synchronous synergies (see section “Materials and Methods”) and corrupted them with both types of noise (*a* = 0.1 and *v* = 1). After extracting muscle synergies from the simulated datasets, we obtained the VAF and decoding curves reported in Figures 2C–E. In all three cases, the ground-truth sets of synergies that explain the task-discriminating variations were correctly individuated, indicating that our method reliably detects synergy sets independently of the precise number of synergies.

***Varying the sources and levels of noise.*** We then estimated the effect of varying the amount of the two types of physiological noise. In these further investigations, we generated the data using all 5 simulated synchronous synergies (plotted in Figure 2A). In particular, we tested how noise differentially affects the selection of synergies with our decoding method and with standard VAF criteria. (The threshold for the VAF-T criterion was set to 0.9 in all subsequent simulations).

We started by generating an EMG dataset with a set of “reference” values of the noise parameters (*a* = 0.1 and *v* = 0.1) on which we performed the subsequent synergy-identification and decoding analysis. Figure 2E shows that the decoding method correctly identified the number of synergies expressing task-discriminating variance. This already suggests that the decoding metric is quite robust to the presence of task-irrelevant variance in the data set.

In the subsequent simulations, we increased the level of one noise parameter at a time in order to gain some intuition about the dependence of the VAF and percent correct metrics on these different sources of noise and also examine the robustness of our method to higher levels of noise.

First, we examined the impact of increasing the signal-dependent noise. We increased the signal dependent noise parameter to *a* = 0.3 keeping *v* unchanged. The VAF drastically decreased indicating its susceptibility to the unstructured additive noise (Figure 2F). In fact, in the limit of very large additive noise (e.g., due to very noisy recordings), the VAF curve exhibited an almost linear increase with low slope implying that a large number of synergies would be required to explain the variance of the dataset (see e.g., Figures 2F,H). These findings highlight a degree of arbitrariness in selecting synergy sets by setting thresholds purely based on VAF criteria, as VAF contains also noise variance and thus choice of a good threshold crucially depends on the amount of noise in the considered dataset. More specifically, in the high additive noise case, the VAF-based criteria select a very large number of synergies because the curve neither reaches the 0.9 threshold nor plateaus. On the other hand, the percent correct metric exhibited robustness to additive noise demonstrating only a slight decrease (Figure 2F). Hence, by attempting to decode the task performed, we can discount task-unrelated variance and we can still identify the synergies that account for task-discriminating variability in a general way and separate them from those explaining unstructured noise in the data.

Second, increasing signal-dependent noise (*v* = 0.3) does not change the VAF curve (Figure 2G)- meaning that the whole dataset can still be reconstructed with low error- but produces synergies whose activations are more variable across trials, i.e., the motor tasks are performed in a less stereotyped way, which causes the decoding performance of the extracted synergies to decrease. However, although the decoding ability of the extracted synergies is decreased, our method is still able to detect the four synergies that explain all the task-relevant variability in the dataset (Figure 2G). Note that in the limit of very large motor noise, decoding would tend to chance level while the VAF would still be near 100% (see e.g., Figure 4C). In such an extreme case, our decoding algorithm would correctly detect that the synergies (though they explain the variance of the data) cannot be used for identifying and selecting the task-discriminating movement features. In fact, the mapping between synergy activations and tasks executed would be completely random, proving the non-usability of this synergistic structure for accurate motor control.

Finally, we increased both types of noise simultaneously (*a* = 0.3 and *v* = 0.3). Consequently, both curves fell to lower values. However, although the VAF curve did not exhibit any elbow or saturation point, the decoding curve had a clear peak at *N* = 5 demonstrating the robustness of our approach to high levels of any type of variability (Figure 2H).

**Figure 5:**
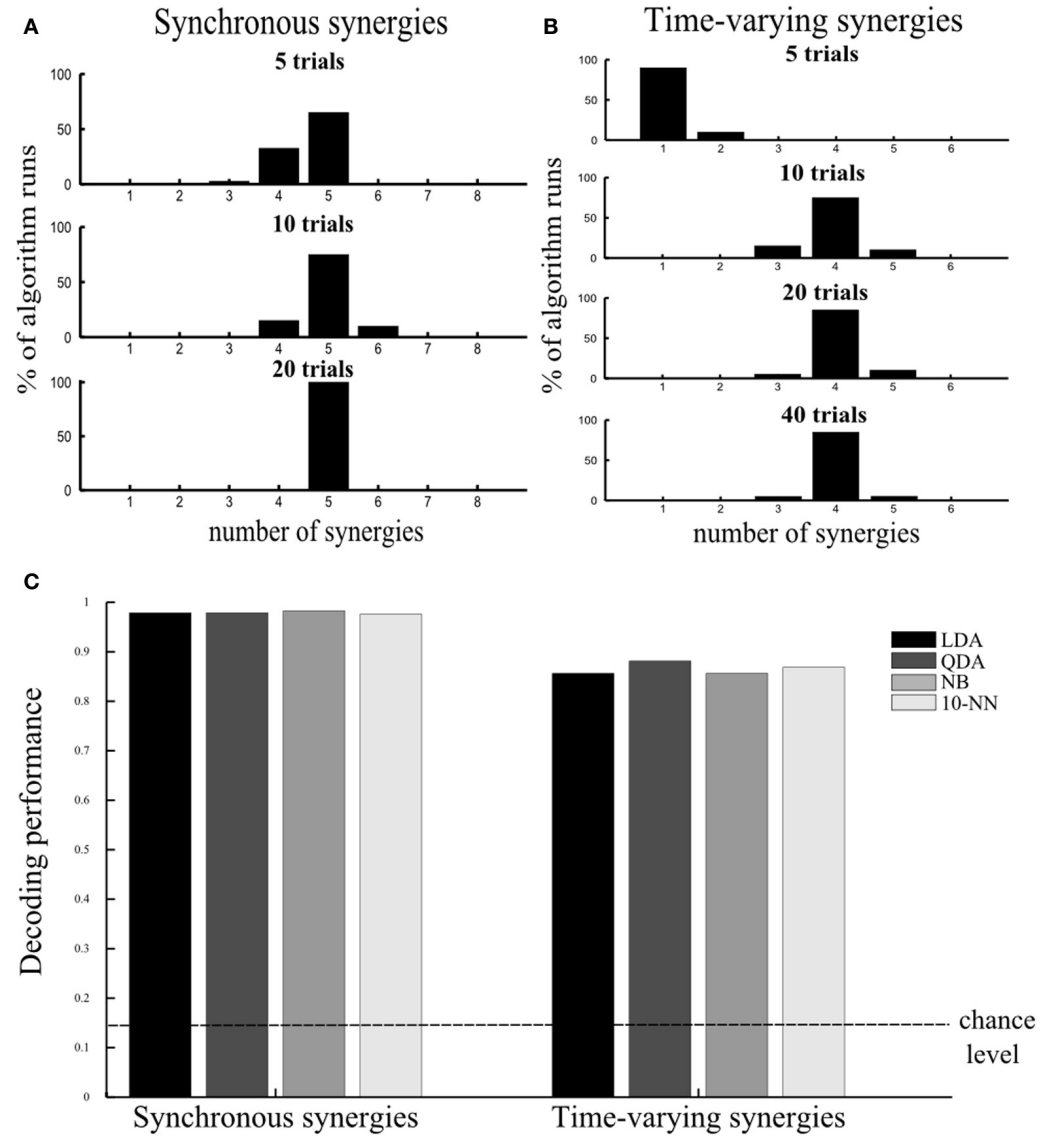
**Assessing robustness of results. (A,B)** Assessing the number of repetitions per motor task required to guarantee robustness of our method. Histogram of the distribution of number of selected synergies across 20 algorithm runs using 5, 10, 20, and 40 trials per task (plotted from top to bottom) for the synchronous **(A)** and the time-varying synergies **(B)**. **(C)** Robustness of results to choice of single-trial decoding algorithm. Histogram of the percent correct values computed using four different decoding algorithms for the synchronous (left) and the time-varying synergies (right). The discrimination power of the selected synergy set was preserved across all decoding algorithms. In all tests, we used the “reference” noise values (*a* = 0.1,*v* = 0.1).

***Varying the number of samples.*** Because the datasets that can be obtained experimentally are composed of a limited number of trials, we investigated how this limitation may affect the performance of our method. Our aim was to assess the smallest number of repetitions of each motor task that can guarantee reliable selection of the set of muscle synergies that account for all the task identification power. In these simulations we used the same noise levels as the ones in Figure 2E (*a* = 0.1 and *v* = 0.1). We generated 5 repetitions of each simulated motor task, i.e., 5 trials × 8 tasks = 40 samples, and corrupted them with 20 different instances of noise yielding 20 different simulated datasets. On these datasets we implemented our decoding-based method and computed how many times it gave the correct result (five). We repeated the same when simulating 10 and 20 repetitions of every motor task. Figure 5A shows that 10 trials per task were sufficient to determine the five synergies in the vast majority of the algorithm runs, while when 20 trials were used the algorithm identified the number of synergies always correctly. In general, we found (results not shown) that increasing the number of samples caused a reduction of both the variance of the decoding performance at fixed number of synergies and of the confidence intervals of the “null hypothesis” (indicated as a shaded red area in Figures 2, 3), which in turn both contribute to increasing the stability of the method. In the case of five trials, although the correct number was found in most cases, sometimes the algorithm underestimated the number of underlying synergies because relying on only 5 trials per task rendered our test of statistical significance not reliable. So, the synergies contributing the least to describing task-related differences were often falsely excluded.

***Using different decoding algorithms.*** To validate that our results do not depend on the specific choice of single-trial decoding algorithm (LDA) we applied our methodology to the simulated dataset constructed from the five synergies (Figure 2A) corrupted with the “reference” noise values (*a* = 0.1 and *v* = 0.1) and 40 simulated trials per task using a wider range of classification algorithms (see section “Materials and Methods” for details). We used these noise levels and number of trials because they reflect the corresponding values used/computed in our experiment. We tested other three standard decoding algorithms (QDA, NB, and 10-NN) and found that all of them identified correctly the set of (five) synergies describing all information about the task. Moreover, the discrimination power of the synergy decomposition was revealed to be robust to the assumptions underlying the classification procedure as the decoding performance was almost identical for all algorithms (Figure 5C left) (also when we varied the number of trials and levels of noise, results not shown).

#### Extension of the method's applicability to time-varying synergies

So far, we validated our method on EMG data described by synchronous synergies. However, it is readily applicable to any type of representation of muscle activation patterns. To demonstrate its robustness independently of the underlying model, we repeated all the tests performed above with simulated data generated from four physiologically plausible time-varying synergies (see section “Materials and Methods”). The reference values of the noise parameters *v* = 1 and *a* = 0.1 used in these simulations were set so that the amount of task-discriminating and non-discriminating variability in the simulated data matched those measured in the experimental data based on which we built the simulations. We note that in all cases adding realistic amounts of noise—as the ones expressed by the parameter values used in all subsequent simulations—allowed accurate reconstruction of the shape of the underlying synergies (shown in Figure 3A).

First, we validated that the method identifies correctly the set of synergies that generated all task-to-task differences independently of the original synergy set dimensionality (Figures 3C–E). Then, using all four synergies, we examined the method's robustness to higher levels of both types of variability. Again, the number of synergies explaining all task-discriminating variations was reliably specified (Figures 3F–H). The slightly larger confidence intervals of the “null hypothesis” (shaded red area) result from the larger variance of the estimates of this decomposition with respect to the synchronous synergies. This can arise from the non-guaranteed convergence of the time-varying synergy extraction algorithm and its sensitivity to the initial guess, two factors that make the output of time-varying decompositions less stable across simulations.

Following this, we aimed at estimating the minimal number of trials required to guarantee reliable synergy set selection. Five trials appeared to be too few for our proposed test of statistical significance (Figure 5B). In this case, the assessment of the number of synergies was not reliable because the distribution of the shuffled surrogates had a very big variance. As a result, any increase of dimensionality did not increase significantly (with respect to the shuffled surrogates) the decoding performance and thus, our method yielded usually one synergy. Therefore, we suggest a minimum of 10 repetitions of each motor task for potential future studies evaluating the importance of trial-to-trial variability in time-varying synergy extraction.

We note that the minimum of trials required to individuate correctly the synergy set may depend upon both the number of muscles recorded by the EMG setup and the level of noise in a current session. The number of trials needed to identify the synergy set is likely to increase with the number of recorded muscles (because decoding in a higher dimensional space with small dataset is more difficult) and with the level of noise (due to the difficulty in detecting patterns in noisy conditions). Whereas our simulations demonstrate that our method can detect reliably the synergy set with feasible amounts of data, for the reasons indicated above it is valuable to evaluate the minimum number of trials at a preliminary stage using simulated data (such as those considered here) constructed with statistical properties as similar as possible to the actual experimental data of interest.

Finally, we examined whether other decoding algorithms yield the same results for the time-varying synergies too (Figure 5C right). Indeed, both the identified number of synergies and their decoding performance were robust once more indicating the applicability of our method independently of the properties of the dataset or the details of the mathematical implementation.

### Identification of the smallest synergy set that accounts for all task-discriminating variability in single-trial synergies extracted during center-out pointing.

To illustrate the ability of our methodology to identify synergy sets on real data, we applied it to a dataset of EMG activity recorded during an experimental protocol (fully described in section “Materials and Methods”) comprising of many repetitions of a variety of point-to-point reaching movements. For each one of the four subjects tested, we formed an EMG matrix of dimensions 9 muscles × (50 time steps × 320 samples) consisting of all the movement-related EMG activity (rectified and filtered) of the 9 muscles for all samples recorded. In order to illustrate our methodology first, we present extensive results from the analysis of only one (”typical”) subject's EMG dataset. In such realistic situation, the correct number of synergies is unknown and the amount of the different types of noise is not available. Here we test the extent to which extracted synergies can express single-trial task-related differences and how many synergies are selected by our method (compared to the VAF-based criteria). A summary of the results of all four datasets is reported at the end of the following section.

#### Synchronous synergy identification

**Figure 6:**
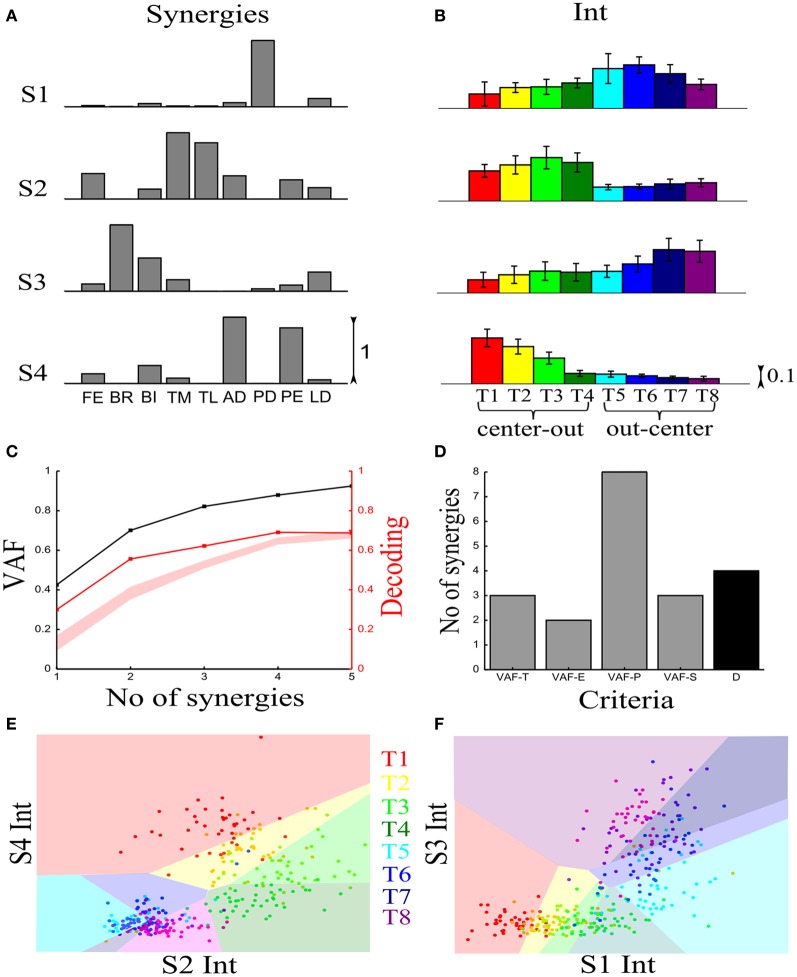
**Application of our decoding method to the synchronous synergies extracted from the EMG data of a typical subject recorded during the execution of an arm pointing task. (A)** The four synchronous synergies obtained from the experimental data of the example subject as four vectors of activation levels of 9 shoulder and elbow muscles. **(B)** Histograms of the integral of the activation coefficient (Int) across the 8 motor tasks performed. **(C)** VAF (black curve whose scale is indicated in left *y*-axis) and percent correct curve (red curve whose scale is indicated in right *y*-axis). The shaded area represents the 5–95% confidence interval of the bootstrap test for decoding. **(D)** Histogram of the number of synergies selected by each one of the existing criteria (gray bars) and our proposed one (black bar). **(E,F)** Decoding the 8 motor tasks using the integral of the 4 synchronous synergies' activation coefficients. For a given trial to be decoded, the activation coefficients of 2 synergies are represented as a point in the 2-dimensional space. The color of each point indicates the actual task which this trial corresponds to. The linear discriminant algorithm has divided the space into 8 regions, one for each class (motor task). The trial is assigned to the class indicated by the color of the region on which the point lies. **(E)** Classification using the integral of activation of synergies S2 and S4. **(F)** Classification in the S1–S3 space.

We first illustrate the application of our method to recorded synchronous synergies. We extracted using the NMF algorithm (see section “Materials and Methods”) single-trial synchronous synergies from the experimental EMG data, beginning with the typical subject (Figure 6). These synergies consist of constant vectors of levels of muscle activations (Figure 6A) recruited by time-varying activation coefficients. Note that the synergies and coefficients shown in this figure were obtained using *N* = 4 in the synergy extraction algorithm because we found (see next sections) that this is the minimal set of synergies explaining all task-discriminating variations. However, the four most task-discriminating synergies and their coefficients obtained assuming more synergies were quantitatively and qualitatively very similar to those presented in Figure 3 (results not shown). The resulting muscle groupings had a straightforward anatomical and functional interpretation: Synergy1 describes mainly the activation of two shoulder flexors; Synergy2 has strong activations of elbow extensors; Synergy3 has strong activations of elbow flexors and Synergy4 is primarily composed of shoulder extensors (Figure 6A).

In order to assess how synchronous synergies allow decoding the task on single trials, we computed the time integral of the synergies' activation coefficients, which represents the magnitude of the synergy activation (see section “Materials and Methods”). The magnitude of each synergy clearly depended upon the task. More precisely, S1 is primarily activated for out-center movements starting from the left; S2 is recruited for center-out leftward movements; S3 is recruited for out-center movements starting from the right and S4 for center-out rightward movements (Figure 6B).

Figure 6C plots the VAF curve as a function of the number of synergies (black curve) and Figure 6D shows the selected number of synergies that would be selected according to each one of these criteria. It is clear that these VAF-based criteria do not yield a consistent number of synergies for this dataset, and selecting the correct set of synergies in this way necessarily relies partly on a somewhat arbitrary choice. We attempted to resolve this problem by complementing the VAF curve with the decoding performance afforded by the single-trial parameters of the model. We applied our proposed decoding-based method on the magnitude of the extracted synchronous synergies. The decoding performance curve (red curve in Figure 6C) obtained by varying the number of synergies (*N*) saturates at *N* = 4 indicating that the task discrimination power added by the magnitude parameters of any additional synergy is negligible. Thus, application of our model selection algorithm gave 4 synergies (last point on the curve lying above the red shaded area).

To gain more insights on how these 8 motor tasks are decoded using the magnitude parameters of the extracted four synergies, we visualized the decoding procedure. In Figures 6E,F, we show scatter plots of the parameters on a 2-dimensional space that has been split by the LDA into 8 different classes, one for each task. Each point is colored according to the actual class which this trial corresponds to and is assigned by the decoding algorithm to the class represented by the colored region on which it lies. Thus, the trial is decoded correctly only if these two colors coincide. From Figure 6E, it is clear that activations of synergies S2 and S4 can discriminate well the forward tasks (T1-T2-T3-T4), but the backward ones(T5-T6-T7-T8) are much less discriminable (larger overlap of the data points). This is why only two synergies are not sufficient to distinguish all the simulated tasks. We may expect that adding the other two synergies will resolve this problem. Indeed, Figure 6F shows that in the S1-S3 space, even though tasks T1-T2-T3-T4 are poorly classified, there is a clear improvement in the classification of tasks T5-T6-T7-T8. So, to get maximal discrimination of the 8 motor tasks four synergies are required, two of them encoding the forward movements and the other two the backward ones.

We then examined the results from the single-trial analysis of the synchronous synergies extracted from all four recorded subjects. The overall decoding performance afforded by synchronous synergies ranged from 63% to 73% for the LDA decoding algorithm across subjects. The set of synergies needed to explain all task-discriminating variability consisted of four synergies in two subjects and three in two other subjects. In general, the synchronous synergies extracted from different subjects were qualitatively and quantitatively similar. The average similarity between the synchronous synergies extracted from different subjects is shown in Figure 8 (lower left part). In the two subjects that needed four synergies to capture all task-discriminating variations, muscle groupings were similar to the ones shown in Figure 6A. In the two subjects that needed only three synergies, the elbow extensors (S2 in Figure 6A) were either grouped with the shoulder extensors (S4 in Figure 6A) to form one synergy or activated by all three remaining synergies at lower levels (results not shown). Hence, S1, S3, and S4 were identified in all subjects, whereas S2 was present only in the two subjects that used four synergies to perform the tasks. In all subjects the decoding performance of the selected synergies was significantly higher-than-random for all tasks (*p* < 0.05, bootstrap test). We also tested the decoding performance of each one of the four synchronous synergies separately and found higher than random decoding for all of them (ranging from 31% to 46%). This implies that each synergy in the set exhibits a degree of tuning to all task directions.

#### Time-varying synergy identification

To characterize the spatiotemporal organization of the recorded muscle patterns, we fed the EMG matrix to the time-varying synergy extraction algorithm and extracted sets of time-varying synergies (see section “Materials and Methods”). Each EMG pattern in each sample was then described by the coefficients specifying the amplitude (scaling coefficient) and time delays of the activation of each synergy. The extracted synergies, the mean activation coefficients and delays across tasks are shown for the typical subject in Figure 3.

**Figure 7:**
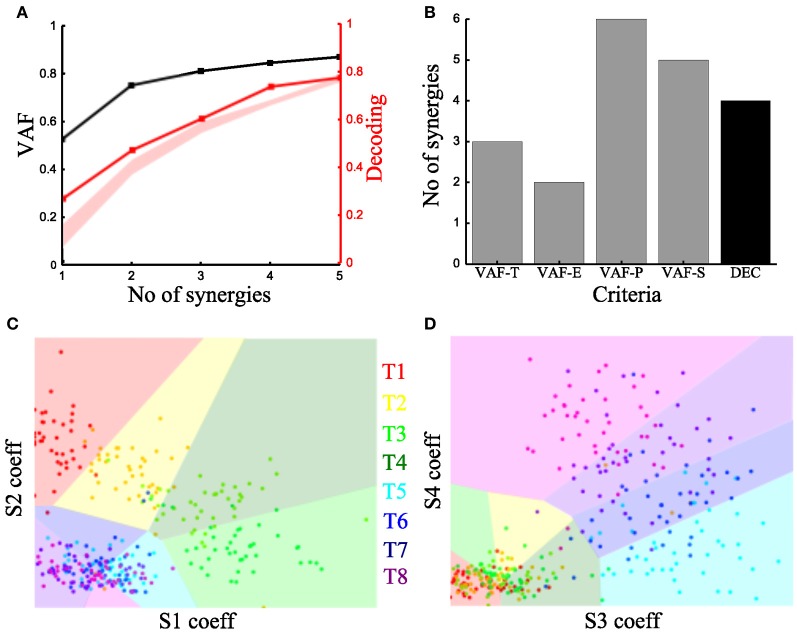
**Application of our method to the time-varying synergies of the typical subject. (A)** VAF (black curve whose scale is indicated in left *y*-axis) and decoding performance curve (red curve whose scale is indicated in right *y*-axis) for the example subject. The red curve represents the percent correct values using the scaling coefficients of the time-varying synergy model. The shaded area represents the 5–95% confidence interval of the bootstrap test for decoding. **(B)** Histogram of the number of synergies selected by each one of the existing criteria (gray bars) and our proposed one (black bar). **(C,D)** Decoding the 8 motor tasks using the 4 time-varying synergies' scaling coefficients. **(C)** Classification using the coefficients of synergies S1 and S2. **(D)** Classification in the S3–S4 space. Region and marker color conventions as in Figures 6E,F.

We then examined how these single-trial parameters were modulated by the task (Figure 3B). We considered the distribution across tasks of the scaling coefficient and found that it exhibited a clear task-dependence (Figure 3B) (D'Avella et al., 2008) similar to the one found for the integral of the synchronous synergies model. The corresponding delays are dependent on the task performed but their relationship with motor tasks is less apparent than that of the coefficients (results not shown). Then, we implemented the decoding-based method opting for the set of time-varying synergies that explain all task-discriminating variability. Our method yielded four synergies (Figure 7A) also for this model. We further asked if we could reach the same result using the VAF-based criteria. Figure 7B shows that each one indicated a different number of synergies rendering such an assessment inconclusive and pointing out the arbitrariness of any selection made using these criteria.

As we did for the synchronous synergies, we examined the discriminability of the motor tasks also in the time-varying synergy activations space (Figures 7C,D). Again, we found the activations of two synergies (S1-S2) describing the differences across the forward tasks (T1-T2-T3-T4) and the other two (S3-S4) were used to distinguish the backward ones (T5-T6-T7-T8).

We note that with respect to previous studies of the role of synergies upon goal-directed reaching movements (D'Avella et al., 2006, 2008), our method individuated in this subject synergies spanning all four cardinal directions in planar reaching space. This may be due to specific subject-to-subject or task setup differences between our experiments and those previously published. Another possibility is that some synergies encoding specific directions carry relatively little EMG variance (such as those pointing right in right-handed movements, e.g., synergy S4 in Figure 3A, which expresses out-center movements starting from the right) and may thus be discarded according to VAF-based criteria. However, our method picks these synergies because they express degrees of freedom of movement relevant for the task and not expressed by other synergies and so must necessarily be included in a task-decoding analysis.

**Figure 8:**
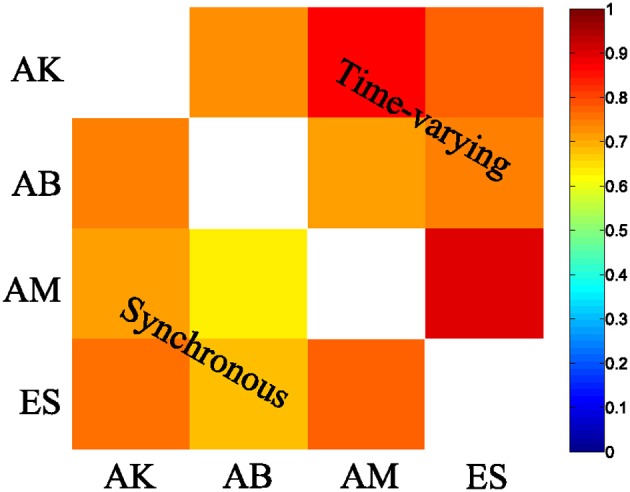
**Synergy similarity matrix across all tested subjects.** The synergy sets extracted from all subjects that were selected by our method exhibited a high similarity either when the synchronous (bottom left) or when the time-varying synergy (top right) model was used.

Similar results held for all four recorded subjects. The overall decoding performance afforded by the scaling coefficients of time-varying synergies ranged from 64% to 74% (for the LDA algorithm) across subjects (for comparison, chance level is 12.5%). The number of time-varying synergies needed to explain all task-discriminating variability was the same as the number of synchronous synergies for all four subjects. The shape of synergies (i.e., the time course and the relative levels of muscle activations) was similar for each subject, as indicated by the high similarity index obtained for all pairs of subjects (Figure 8 upper right part). The main difference across subjects was that in the two subjects that needed only three synergies the muscle activations that constitute the fourth (non-significant) synergy was either included in one of the other three synergies or distributed across all of them (results not shown). The resulting scaling coefficients were modulated accordingly so as to produce movements to all task directions and describe identifiably all motor tasks. Indeed, in all subjects the scaling coefficients of the selected synergies had significantly higher-than-random decoding performance (*p* < 0.05, bootstrap test) for all reaching directions. Furthermore, we found that all time-varying synergies too exhibit some degree of tuning to all task directions (individual decoding performance ranged from 33,75% to 38,75 %).

In sum, a small set of time-varying or synchronous synergies described all task-related differences in the examined EMG datasets. This set of synergies would have been hard to select relying on VAF-based criteria, because some of the standard criteria would exclude synergies that express task-discriminating muscle activations and contribute to the generation of each task, while others would include synergies that explain only task-irrelevant variations reflecting different sources of noise in the recorded data.

### Using the decoding formalism for comparing the ability of muscle synergy models to encode task-discriminating information

We further note that the overall decoding performance of a synergy model may be used as a criterion to decide which type of synergy decomposition is most suitable to describe a set of tasks and which (and how many) parameters of such a representation carry information about the task effected at hand. To illustrate this point, we compared the decoding performance of both time-varying and synchronous synergies with comparable number of parameters per synergy for all subjects tested.

**Figure 9:**
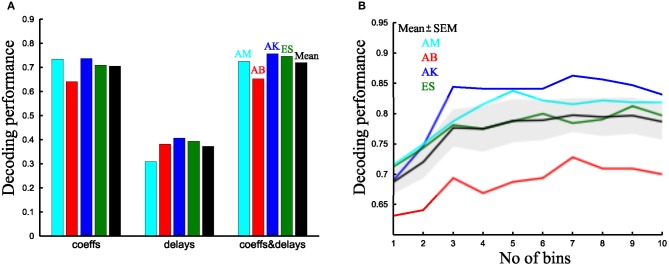
**Comparison of the decoding performance of synchronous and time-varying synergies. (A)** Percent correct values of the time-varying synergy decomposition for all four subjects tested using only the scaling coefficients (left), only the delays (middle) and both single-trial parameters per synergy (right). The last column (black) is the mean across subjects. **(B)** Decoding performance as a function of the number of bins in which the integral of the activation coefficient is split for all subjects. The black curve and shaded area represents the mean ± SEM across subjects.

We first examined decoding performance when using one parameter per synergy. We started by assessing the discrimination power of the two single-trial parameters of the time-varying synergies (i.e., scaling coefficients and time delays). Although decoding with the time delays is significantly above chance level (1/8 = 12.5%) for all subjects, the scaling coefficients afford a significantly higher decoding performance (37.3 ± 4.3% vs. 70.5 ± 2.2% respectively, Figure 9A) (*p* < 0.05, paired t-test). Comparing the scaling coefficients of the time-varying synergies with the integral of the activation coefficients of the synchronous synergies (Figures 9A,B), we found that the time-varying and the synchronous model had comparable performance (70.5 ± 2.2% vs. 69 ± 1.9% respectively). The corresponding VAF values for these synergy decompositions were 81 ± 2.1% vs. 89 ± 1.4% respectively. Consistent with previous findings (D'Avella and Bizzi, 2005), the synchronous synergy decomposition captures a significantly higher percentage of the variability of the data (*p* < 0.05, paired t-test).

Then, we evaluated the decoding performance gain obtained when combining two parameters per synergy. For the time-varying synergies, using both parameters yielded a significantly better decoding than with the scaling coefficients alone (72 ± 2.7% vs. 70.5 ± 2.2% respectively, Figure 9A) (*p* < 0.05, paired *t*-test). For the synchronous synergies, we divided the movement duration in two phases; we computed the integral of the synchronous synergy activation coefficients for each one of them and used these values as the decoding parameters. Also for this model, decoding using two parameters was significantly higher than with one (72 ± 2.6% vs. 69 ± 1.9% respectively) (*p* < 0.05, paired *t*-test). Then, we compared the task discrimination power of the two models. We found that, when using two parameters per synergy (scaling and delay coefficients for the time-varying model and the integral in two equal bins for the synchronous), the decoding performance of both types of synergy decomposition was 72 ± 2.7% vs. 72 ± 2.6% respectively (Figure 9B). Thus, for this particular set of tasks and muscles, both synergy decompositions seem equally adequate and equally compact in describing the task-discriminating variations in muscle activation signals. Indeed, equal decoding performance is obtained when using the same number of parameters in both approaches (*p* < 0.05, paired t-test).

Finally, we tested whether adding more single-trial parameters per synergy adds more information about the task. As the time-varying synergies have only two single-trial parameters, we had to restrict the analysis to the synchronous synergy model. By binning the activation coefficients in gradually shorter time bins and computing the corresponding integrals, we progressively increased the number of parameters used for decoding. Figure 9B depicts the decoding performance for all subjects as a function of the number of bins. Decoding performance saturated quickly for all subjects at approximately three bins, meaning that all task-discriminating information can be described by considering three basic phases of one-shot rapid movements. This is reminiscent of the well-known triphasic pattern observed during the control of ballistic single joint rotations but also during whole-body actions (a first agonist burst followed by an antagonist burst followed by a second agonist burst) (Berardelli et al., 1996; Chiovetto et al., 2010). This result points out that despite the potentially high number of free parameters describing the activation time course of synchronous synergies, the task being performed may be encoded by a much more compressed set of parameters without loss of information about task-discriminating variations in synergy recruitment.

In sum, in this particular dataset the synchronous synergy model was slightly more effective at describing task-discriminating variability than the time-varying one when the time course of synchronous synergies was modeled by three or more parameters. This corresponds to a slight increase in model complexity with respect to the use of time-varying synergies whose single-trial activation is described by two parameters per synergy. However, when using the same number of parameters, time-varying and synchronous synergies explained the same amount of task-discriminating variability.

## Discussion

In this article we proposed and implemented an automated method, based on single-trial task decoding, to evaluate the extent to which muscle synergy recruitment can be mapped onto motor task identification. We showed how this new metric complements the VAF metric commonly used to test the validity of muscle synergy decompositions and can be used to address questions related to movement execution in task space. Quantifying the degree of validity of muscle synergies in task space is fundamental since synergies (as extracted from dimensionality reduction techniques) are assumed to be the building blocks of movement production that can be reused across tasks, while the synergy combination coefficients represent the task-dependent motor features. This study presents a systematic procedure to measure the degree of feasibility of such a modular and hierarchical control scheme by means of a single-trial task decoding technique. The significance of our conceptual and computational developments is discussed in the following.

### Conceptual foundations of the method

Fundamental to the method is the fact that motor behaviors are produced on single trials and therefore should be analyzed on such a basis (Ranganathan and Krishnan, 2012; Ting et al., 2012). Even though single-trial analysis is not new in the study of muscle synergies (Tresch et al., 1999; Torres-Oviedo and Ting, 2007, 2010; Kutch et al., 2008; Valero-Cuevas et al., 2009; Chvatal et al., 2011), it appears to be a necessary and rational component in our methodology (otherwise task-decoding would be trivial with data averaged per task). Thus, exploiting single-trial analysis tools, we came up with a computational procedure that quantifies the task identification power afforded by different synergy decompositions. Our method uses this formalism to separate out trial-to-trial variations in synergy space that reflect task-discriminating variability (and hence increase decoding performance) from those that do not account for task-related differences. The latter can be regarded as “noise” as far as task discrimination is concerned, even if they reflect neurophysiological processes. This conceptualization mirrors the one often used in single-trial neural literature to separate signal from noise and identify the most informative components of neural responses (Quian Quiroga and Panzeri, 2009). The advantage of this method is that it allows the user to focus on task-discriminant aspects of variability using an objective and useful scale in a user-defined “task space” rather than measuring variability on a scale related only to the amplitude of the EMG signals (Quian Quiroga and Panzeri, 2009; Tolambiya et al., 2011).

As exemplified by our application to real data, one potential advantage of the decoding metric is that it can identify muscle activation components of relatively low amplitude (and accounting for a small amount of the VAF) but reflecting unique information about the task. Furthermore, a comparison of synergy sets determined by VAF or by decoding metrics may be useful to tease apart synergies that provide unique information about task-to-task differences (such as the synergy set individuated by decoding) from synergies that are task invariant but contribute an important part of variance because they e.g., implement muscle activation for maintaining posture. Such important task invariant synergies are likely to appear as “extra” synergies selected by VAF method (because they carry variance) but not from the decoding method (because they do not add much task discriminating power).

Interestingly, in the experimental data we found that our method revealed relatively high decoding scores for relatively small synergy sets, supporting the idea that a small set of synergies are recruited in different ways during the execution of a larger number of different motor tasks. This finding is compatible with the idea of muscle synergies as intermediate low-dimensional representations of the mapping between motor commands and task goals and the associated theory of hierarchical motor control (Ting and McKay, 2007; Todorov, 2009).

The hierarchical view of motor control implies that a desired motor behavior can be mapped onto specific recruitment of muscle synergies whose activation leads to the expected behavior (Bizzi et al., 2000; Tresch and Jarc, 2009). Due to the non-linearities of the musculoskeletal plant, even small variations in the muscle pattern could lead to very different behaviors at the end-effector level and thus could affect task achievement. Therefore, evaluating the effectiveness of synergies in terms of motor task performance is crucial. Ultimately biomechanical modeling/simulation is needed to achieve this (Neptune et al., 2009; Kargo et al., 2010). Task-decoding is a useful additional metric to validate (or invalidate) a certain chosen synergy decomposition before or simply without this step. High task-decoding scores lend credit to a synergy decomposition. In contrast, low task-decoding scores (e.g., decoding performance that is robustly around chance level for different decoding algorithms) may falsify a given synergy decomposition, even if VAF is close to 100%. Generally speaking, the decomposition/model yielding concurrently the highest VAF and the highest decoding score could be viewed as the most likely representation of neural synergies implemented in the CNS. In practice, the decoding metric was shown to be more robust to various sources of noise than the VAF. As such, it provides a more stable reference to inter-subject or inter-study comparisons. Concretely, this approach can be useful to reduce side effects on VAF values related to the different material, subjects or signal pre-processing used by different researchers.

Previous studies have proposed to separate task-discriminating from non-discriminating variations by evaluating VAF within each task separately (Torres-Oviedo and Ting, 2010; Chvatal et al., 2011; Roh et al., 2011) or identifying task-specific muscle synergies (Cheung et al., 2009a). These methods are very effective when some tasks in the examined set are executed using synergies that are not shared with other tasks. On the contrary, our method is more effective when muscle activations in different tasks differ not because of the highly specific activation of synergies only in particular tasks, but rather because of different activation coefficients of the same group of synergies. In this latter case, application of synergy decompositions separately in each individual task would lead to the identification of a larger set of synergies than those actually generating the task-discriminating muscle activations (we verified this intuition also by extracting synergies in one task at a time in our simulated datasets, which invariably led to incorrect identification of a larger set of synergies with lower task-discrimination power than the synergy set determined by our method; data not shown).

Our findings parallel results recently obtained in frogs' experimental and modeling studies (Kargo and Giszter, 2000, 2008; Kargo et al., 2010). In these studies, the authors varied the initial configurations as well as the level of muscle vibrations applied to the frog's hindlimb and showed that flexible combinations of a small set of invariant muscle patterns can produce successful accurate targeting of the frog's hindlimb from a large range of starting positions. It would be of interest to apply our method to this dataset in order to assess quantitatively the reliability of the correspondence between such task variables or feedback stimuli and muscle pattern activations. We believe that our method could serve to determine the functional role of the identified muscle patterns and further evaluate the significance of their coupling under different experimental conditions.

### On the computational method and its use

#### Critical comparison of the validity of different synergy models

An advantage of the derived decoding metric is that it allows a direct comparison of performance of different synergy decompositions in terms of task execution when using the same number of parameters in all decompositions. This is useful for testing the validity of various hierarchical motor control schemes or various mathematical representations of muscle synergies. For example, the synchronous synergy model may account for more variance than the time-varying synergy one (as happens in the EMG dataset considered here) but this may be due to the fact that synchronous synergies are potentially characterized by a larger number of parameters than time-varying synergies (because the former report the full time course of activation, whereas the single-trial activation of the latter is described by two parameters per synergy) (D'Avella and Bizzi, 2005; D'Avella et al., 2006). Specifically, by applying this metric to our dataset we found that the two decompositions decode tasks equally well when using the same number of parameters. Moreover, our formalism can be used to evaluate the loss of task-discriminating information due to simplification of the time course of synergy activation. In our example dataset, our analysis suggested that a full representation of the temporal pattern of activation patterns of synchronous synergies is not crucial to encode the goal of the task. Only the average activation during two or three temporal phases seems sufficient to know what target will be reached.

To shed more light onto the effectiveness of synergies as low-dimensional structures for motor control, future research could aim at extending our formalism to quantify the predictability of kinematic movement features in continuous time (Corbett et al., 2010) rather than just decoding which task (out of many) was performed. This would enable a proper comparison between the dimensionality of the instantaneous kinematics needed to perform tasks and the dimensionality of the muscle representations that explain all the kinematic range elicited by task execution. Furthermore, it would be of interest to apply our methods to experimental datasets containing a wider range of complex movements to determine which synergy decomposition is more effective in general and particularly in more “daily-life” situations. For example, in an entire reach-and-grasp motor task, we could expect that the time-shifts of the time-varying synergy model or a more detailed consideration of the temporal profile of the synchronous synergy activations would be more relevant to task discrimination.

#### Automated selection of the minimal number of synergies

Since our framework allows critically evaluating any muscle synergy model, it can be used in particular to select the minimal number of synergies at fixed synergy representation. Assuming, for example, time-varying synergies as the model from which movements are generated, the simple question of how many synergies are required to describe distinctly the entire set of motor tasks under consideration is usually difficult to answer. The empirical and intuitive VAF-based methods are not automatic or lack objective rationale. Even more problematic is the fact that a synergy accounting for a large or small amount of the total VAF might have no implication with respect to the task goal. We proposed a recursive and automated method to compare synergy set of *N* elements with synergy set of *N* − 1 elements. The basic argument is that if adding a synergy improves significantly task decoding performance then increasing the dimensions by one is worthwhile. We showed that this procedure is effective on simulated data sets for various levels of noise. However, examining decoding alone might be insufficient in some cases: for some data sets, one synergy could allow perfect decoding even though the muscle pattern cannot be reconstructed accurately (very low VAF). To cope with such cases, a potential variation of our method may imply choosing first a lower bound for *N* based on an inspection of the VAF graph, and then running our automated procedure from this *N* to refine the selection of the number of synergies. More generally, considering both VAF and decoding seems important to fully understand he function of the computed synergies, as a consequence of the above discussed complementarity of the two metrics.

### Possible applications and extensions

There is evidence that the activity of motor cortical neurons in the monkey (Holdefer and Miller, 2002) and the cat (Yakovenko et al., 2010) during reaching movements as well as spinal interneurons in the frog during reflex motions (Hart and Giszter, 2010) correlates with muscle synergies and/or their recruitment. These studies suggest that the physiological basis of muscle synergy structures, which is observed in the motor output and assumed to simplify motor control, may rely on a focused selection of a set of neurons. We note that our decoding approach could be in principle applied to decode from single-trial neural population patterns activating synergies, to determine which patterns encode the task and which patterns carry additional or independent information to that carried by other patterns. Application of our method to simultaneous recordings of neural activity and EMGs during the execution of different movements could therefore lead to the determination at the same time of the minimal set of synergies and the minimal set of neural activity patterns that explain all task-discriminating neural and muscle activity, and to the specification of an explicit link between these two sets. These considerations suggest that the work presented here lays down the foundations for a deeper understanding of the relationships between single-trial neural activity and the resulting recruitment of muscle synergies.

More generally, this investigation could reveal itself useful for human-machine interfaces and neuroprosthetics (Nazarpour et al., 2012; Ting et al., 2012). Assuming it is possible to decrypt movement intention (e.g., what target a subject wants to reach to), a set of synergies could be recruited in accordance and, if our metric showed good task-decoding performance of the synergy decomposition considered, we could ensure that a coordinated multijoint arm movement would be generated to the expected target. Recent works aimed at exploiting synergies to control arm and hand motion (Jackson et al., 2006; Radhakrishnan et al., 2008; Vinjamuri et al., 2011) and assessing the validity of muscle synergies in task space appears to be a basic prerequisite for the effectiveness of such techniques.

### Conflict of interest statement

The authors declare that the research was conducted in the absence of any commercial or financial relationships that could be construed as a potential conflict of interest.
